# Evaluation of fluralaner and afoxolaner treatments to control flea populations, reduce pruritus and minimize dermatologic lesions in naturally infested dogs in private residences in west central Florida USA

**DOI:** 10.1186/s13071-016-1654-7

**Published:** 2016-06-28

**Authors:** Michael W. Dryden, Michael S. Canfield, Kimberly Kalosy, Amber Smith, Lisa Crevoiserat, Jennifer C. McGrady, Kaitlin M. Foley, Kathryn Green, Chantelle Tebaldi, Vicki Smith, Tashina Bennett, Kathleen Heaney, Lisa Math, Christine Royal, Fangshi Sun

**Affiliations:** Department of Diagnostic Medicine/Pathobiology, Kansas State University, Manhattan, KS 66506 USA; Animal Dermatology South, 7741 Congress St, New Port Richey, FL 34653 USA; Merck Animal Health, 2 Giralda Farms, Madison, NJ 07940 USA

**Keywords:** *Ctenocephalides felis felis*, Cat flea, Dogs, Field study, Fluralaner, Flea, Flea control, Afoxolaner, Flea Allergy Dermatitis, Flea Bite Dermatitis, Pruritus, Atopic Dermatitis

## Abstract

**Background:**

A study was conducted to evaluate and compare the effectiveness of two different oral flea and tick products to control flea infestations, reduce pruritus and minimize dermatologic lesions over a 12 week period on naturally infested dogs in west central FL USA.

**Methods:**

Thirty-four dogs with natural flea infestations living in 17 homes were treated once with a fluralaner chew on study day 0. Another 27 dogs living in 17 different homes were treated orally with an afoxolaner chewable on day 0, once between days 28–30 and once again between days 54–60. All products were administered according to label directions by study investigators. Flea populations on pets were assessed using visual area counts and premise flea infestations were assessed using intermittent-light flea traps on days 0, 7, 14, 21, and once between days 28–30, 40–45, 54–60 and 82–86. Dermatologic assessments were conducted on day 0 and once monthly. Pruritus assessments were conducted by owners throughout the study. No concurrent treatments for existing skin disease (antibiotics, anti-inflammatories, anti-fungals) were allowed.

**Results:**

Following the first administration of fluralaner or afoxolaner, flea populations on pets were reduced by 99.0 % and 99.3 %, respectively within 7 days. Flea populations on the fluralaner treated dogs were 0 (100 % efficacy) on days 54–60 and 82–86 after the administration of a single dose on day 0. Administration of 3 monthly doses of afoxolaner reduced flea populations by 100 % on days 82–86. Flea numbers in indoor-premises were markedly reduced in both treatment groups by days 82–86, with 100 % and 98.9 % reductions in flea trap counts in the fluralaner and afoxolaner treatment groups, respectively. Marked improvement was observed in FAD lesion scoring, Atopic Dermatitis lesions scoring (CADESI-4) and pruritus scores with both formulations.

**Conclusions:**

In a clinical field investigation conducted during the summer of 2015 in subtropical Florida, a single administration of an oral fluralaner chew completely eliminated dog and premises flea infestations and markedly reduced dermatology lesions and pruritus. Three monthly doses of the afoxolaner chewable also eliminated flea infestations in dogs, markedly reduced premises’ flea populations and similarly improved dermatology lesions and pruritus.

## Background

Fleas are an extremely important blood sucking parasite that infest dogs and cats worldwide. Flea infestations are medically and economically important because fleas are not just irritating to dogs and cats, they can produce anemia, allergic dermatitis, carry several bacterial pathogens, and serve as the intermediate host for cestode and filarid parasites [1].

A number of field studies conducted in Australia, Europe and the United States have documented that a variety of modern topical and oral flea products can effectively eliminate flea infestations [2–14]. Compounds such as afoxolaner, dinotefuran-pyriproxyfen, fipronil (±, (s)-methoprene) imidacloprid, indoxacarb, fluralaner, lufenuron (+pyrethrin spray or + nitenpyram tablets), selamectin, and spinosad have been found in these various studies to be effective in reducing or eliminating flea infestations on naturally infested dogs and cats without the need for premises treatments [2–14].

Fluralaner and afoxolaner are recently introduced oral flea and tick adulticides in the isoxazoline class of drugs. Both drugs work as GABA-Chloride antagonists causing over excitation of the insect and arachnid nervous system and rapid ectoparasite death [15, 16]. Both molecules have demonstrated rapid and persistent efficacy against fleas and multiple species of ticks [17, 18].

Following the administration of a fluralaner chew, efficacy has been maintained against fleas in both field and laboratory studies for 12 weeks [14, 19, 20]. A single dose of a fluralaner chew has been shown to start working within 2 h of administration, eliminating 88 % of an existing flea population on dogs within 4 h [19]. In a separate study fluralaner killed newly acquired female fleas rapidly enough that no eggs were laid after repeated infestations for 120 days [21]. In multicentric field studies evaluating dogs not managed with associated medications, fluralaner’s rapid and pronounced efficacy against fleas improved clinical signs associated with Flea Allergy Dermatitis (FAD) by 80–96 % [14] and 85.7 % [20]. In those studies, the veterinarian’s experience was used to determine if the signs were consistent with FAD. Recently, two more controlled studies using fluralaner were performed showing 98.8 % and 100 % resolution of FAD signs within 12 weeks, respectively in 20 dogs diagnosed with FAD [22, 23]. In one study clinical signs of FAD were based upon clinical examination, and a positive response on serologic and intradermal evaluations of flea allergen [23]. The other study was an open pre-treatment versus post-treatment assessment, where clinical signs of FAD were clinically evaluated by a veterinary dermatologist [22].

Afoxolaner has an onset of activity of 4 h against fleas [24] and has been shown to reduce flea egg production by greater than 99 % for one month [25]. A laboratory study comparing one dose of fluralaner and 3 doses of afoxolaner administered 28 days apart showed a statistically significant difference in flea adulticide efficacy between products at 6 and 12 h post-infestation time points on days 70 and 84. Throughout the 84 day study there was no statistically significant difference in flea reductions between products when assessed at 24 h after each weekly infestation with efficacies of 99.6 % and 100 % respectively on day 84 [26]. The flea reduction efficacy of afoxolaner administered monthly to client owned dogs in Tampa, FL was 100 % within 6 weeks [11].

The objective of this study was to evaluate and compare the efficacy of fluralaner and afoxolaner oral treatments against fleas on naturally infested dogs in subtropical Florida using both premises and on-animal flea population estimating techniques. Additionally, this study was designed to determine the effect of these treatments on reducing flea associated dermatitis and pruritus. The methodology used in this investigation eliminated client compliance and adherence problems because investigators administered products to all animals.

## Methods

### Home and pet study inclusion criteria

Through referrals from Sunshine Animal Hospital, Tampa, FL, Animal Dermatology South, New Port Richey, FL, and advertisements on CRAIGSLIST®, 36 private residences were selected for inclusion in the study from May 19 - June 08, 2015.

Homes were selected based on the following criteria: 1) a minimum of five fleas observed in area flea counts on at least one dog at the residence; 2) a minimum of five fleas collected in a 16–24 h period in two intermittent light flea traps; 3) one to five healthy, non-fractious dogs at the residence (no cats); 4) qualifying dogs must spend > 50 % of their time in the indoor premises; 5) homeowner’s willingness to participate in the 3 month study; 6) owners agreeing not to use any other topical or premise flea control products during the study and no history of residual topical or oral flea products used in the previous 30 days; 7) owners agreeing not to bring any other mammalian pets into the household for the duration of the study; 8) no pregnant or nursing dogs in the household; 9) dogs qualifying for the study must be > 6 months of age and > 4.4 lb; 10) completion of a questionnaire concerning pet habits, visiting pets, previous flea treatments and personal observations around their residence concerning wildlife and feral cats and 11) owners willingness to sign an informed consent form.

### Treatment groups

Homes and dogs meeting these criteria were placed into 1 of 2 treatment groups. Home entry numbers (1–36) were each assigned a random number by Excel (Excel 2013) and blocked into groups of 2. The highest random number within each block was assigned to group 1 and lowest to group 2.

Dogs in treatment group 1 were administered an oral fluralaner chew (Bravecto®, Merck Animal Health) once on day 0 according to label dosing recommendations.

Dogs in group 2 were administered an oral afoxolaner chewable (NexGard® Chewables, Merial) according to the label dosing recommendations 3 times during the 82–86 day study: once on day 0, once between days 28–30 and once again between days 54–60.

All dogs were weighed prior to each treatment and products were administered by members of the Kansas State University (K-State) Flea Team who were not blinded to treatment groups. While only dogs meeting the inclusion criteria were included in the study for data collection, all dogs living at a residence were administered group appropriate treatment. No other on-animal or oral flea product or premises flea treatments were used during the 12 week study. There were no restrictions on the animals regarding exposure to rain, swimming, bathing, or movement outdoors.

This study was conducted without a placebo control group. While the use of a non-treated group might have provided a better evaluation of the performance of the two treatment regimens, it is the opinion of these authors that the large flea infestations commonly encountered in the Tampa, FL area preclude the use of a non-treated group. Withholding treatment would be detrimental to the health and welfare of both the dogs, and potentially even the humans in a household.

### Flea population assessment

The numbers of adult fleas present in the indoor premises were assessed using intermittent light traps [3–8, 10, 11, 27, 28]. One trap was placed in each of two rooms for 16 to 24-h. Rooms were selected based on where the dog(s) spent most of the time or where owners had observed fleas. Once rooms were selected, the traps were returned to the same rooms in the same location at every counting period. Fleas collected on the adhesive pads of the traps were enumerated and identified by microscopic observation as to determine species.

The flea population on each pet was assessed using a visual area count methodology [3–8, 10, 11, 29]. Area counts were performed at five locations on each animal; dorsal midline, tail head, left lateral, right lateral, and inguinal region. Area counts were limited to one minute per location and conducted by parting the hair against the lay using both hands until the area was covered. Maximum number of fleas per zone was capped at 50; therefore, the maximum total area flea counts for a dog was 250. Area (on-animal) and environment flea counts were conducted ± 1 day on days 0, 7, 14, 21, then once between days 28–30, 40–45, 54–60 and 82–86. Personnel conducting pet and premises flea counts were not blinded to treatment groups.

## Evaluation of skin disease and pruritus

**Fig. 1:**
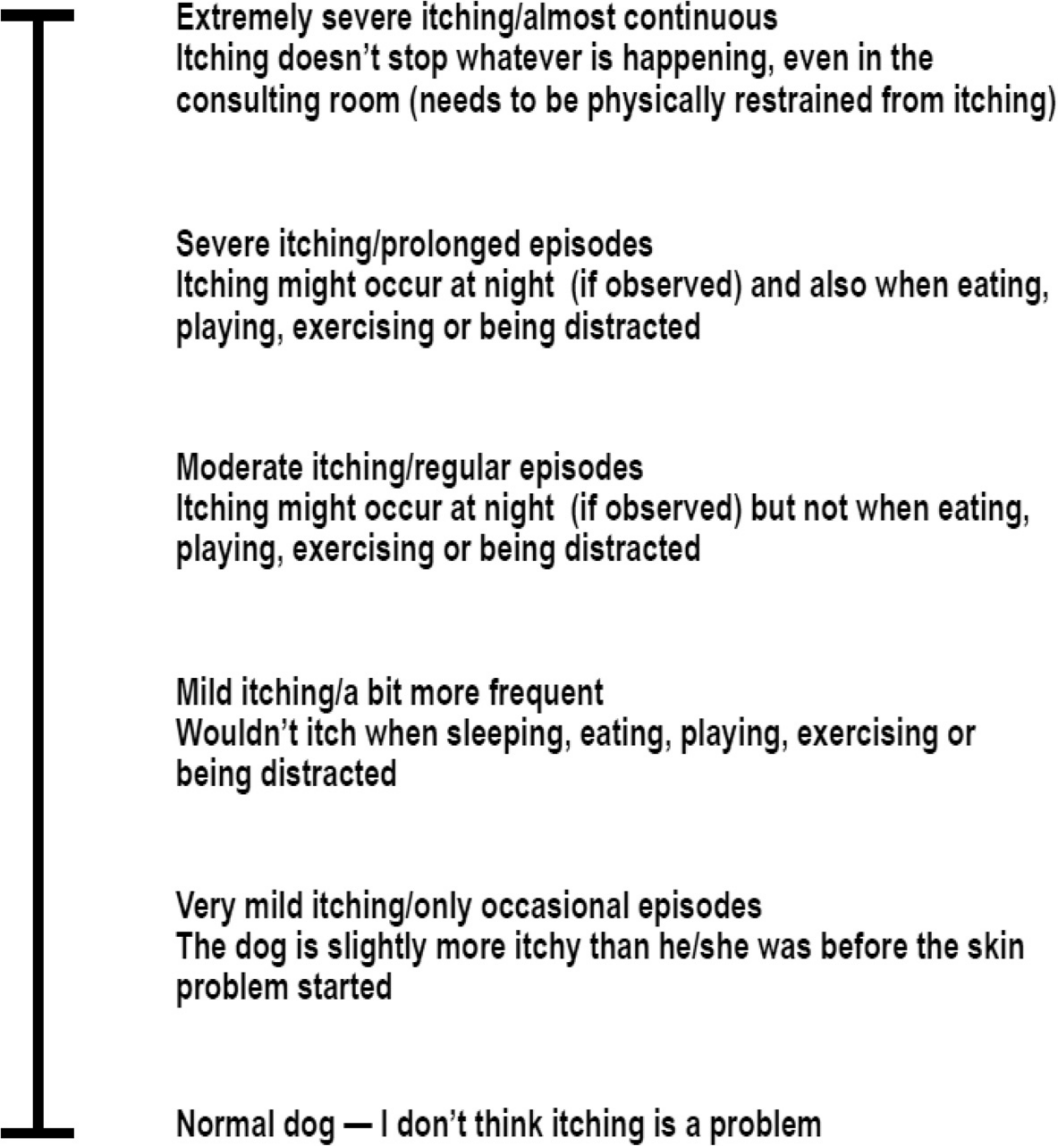
Pruritus Visual Analogue Scale

At visits occurring on days 0, 14, 28–30, 40–45, 54–60, and 82–86, the owner was asked to rate the pruritus level of the qualifying dog(s) in the home on a non-numeric scale using a data capture form. This form provides 6 written descriptions of increasing pruritus severity, from “Normal dog—I do not think itching is a problem” through “Extremely severe itching…” (Fig. 1) [30, 31]. Following the rating of pruritus by the owner, a numeric scale of 0–10 was placed on the form and a numeric assessment of the pruritus level was recorded. Owners did not see and were not informed of the numerical scale or their dog(s) score during the study. In a household with more than one owner, only one owner was allowed to assess the pruritus level of the dog(s) throughout the study.

Masked as to treatment groups, clinical dermatologic observations were made on days 0, 30, 60, and 84 of the study (±3 days) of all qualifying dogs in the homes. To ensure repeatability of results, a single Board-certified veterinary dermatologist (MC) made all of the observations. To assess skin lesions two different systems were used. Dermatologic signs potentially associated with Flea Allergy Dermatitis were assessed using a flea bite hypersensitivity severity scale that evaluated erythema, papules, crusts, scale, alopecia, and excoriation [32]. Each of these categories was graded by the board-certified veterinary dermatologist using a scale from 0 to 3, 0 = no signs, 1 = mild, 2 = moderate, 3 = severe. Three body sites were assessed, (1) dorsum, from the withers to the base of the tail, (2) left and right lateral thorax, just caudal to the elbow and extending to the last rib, (3) “flea triangle”, including the dorsal lumbosacral region, caudomedial thighs, and ventral abdomen (Table 1) [32]. Scores for each of the six dermatology categories, for each body site, and the total sum of all of these sites and conditions were calculated for each dog at each observation. Additionally, dogs were also assessed by the dermatologist using the canine atopic dermatitis (CAD) extent and severity index scoring system (CADESI-4) (Table 2) [33], as a means to assess the skin in regions more commonly thought to be abnormal in atopic dermatitis patients. There is, however, overlap in the two scoring systems.

**Table 1:** Flea bite hypersensitivity severity scoring form

	Site 1 (Dorsum)	Site 2 (Lateral Thorax)	Site 3 (Flea Triangle)	Sum of all sites and categories
Erythema				
Papules				
Crusts				
Scale				
Alopecia				
Excoriation				
Total scores				

Scale:

0 – no signs

1 – mild

2 – moderate

3 – severe

**Table 2:** Canine Atopic Dermatitis Extent and Severity Index-4 (CADESI-4) scoring form

			Erythema	Lichenification	Excoriations and/or Alopecia	Total
Perilabial Area (left and right combined)		1				
Medial Pinnae (concave pinnae)	Left	2				
	Right	3				
Axillae	Left	4				
	Right	5				
Front Paws (dorsal and palmar sides combined)	Left	6				
	Right	7				
Hind Paws (dorsal and palmar sides combined)	Left	8				
	Right	9				
Cubital Flexor (elbow folds)	Left	10				
	Right	11				
Palmar Metacarpal (from carpal to metacarpal pads)	Left	12				
	Right	13				
Flanks	Left	14				
	Right	15				
Inguinal Areas (groin)	Left	16				
	Right	17				
Abdomen		18				
Perineum (from vulva/scrotum to anus)		19				
Ventral Tail (proximal)		20				
Grade each site and each lesion type: None: 0; mild: 1; moderate: 2; severe: 3;			Total score (20x3x3=180)			

### Data analysis

The dog and trap counts were analyzed separately at each time point, using the two-sample *t* test to test for differences in the two groups. Statistical analysis was performed using SAS version 9.1 or higher. The animal and trap flea count data were transformed prior to analysis using the Y = log (x + 1) transformation.

The log transformed flea counts on dogs were analyzed by a mixed linear model with repeated measure including treatment, day, treatment by day as the fixed effects; and household, and animal as random effects. The log transformed flea counts in traps were analyzed by a mixed linear model with repeated measure including treatment, day, and treatment by day as the fixed effects and household as random effect.

A Kenward-Rogers adjustment was used to determine the denominator degree of freedom for hypothesis. Akaike’s Information Criterion (AIC) was used as the criterion to select the covariance structure for repeated measures.

The dermatology, pruritus and CADESI scores were analyzed by the same mixed linear model with repeated measure as that for the flea counts on dogs.

Percentages of animals without fleas and the percentages of homes without fleas (defined as homes where no fleas were recovered on either traps or dogs), were analyzed and compared.

All comparisons were made between treatment groups on each data collection day and also between each collection day and the baseline (Day 0) values within each treatment group.

A two-tailed significance test was used for the comparison and significance was declared when *P <* 0.05 and 90 % confidence intervals were constructed for the differences between treatment groups for the equivalence declaration.

The primary software was SAS version 9.3 (SAS Institute Inc., Cary, NC, USA).

Percent control of fleas counts were calculated using geometric means with Abbott’s formula:

$$ \mathrm{Efficacy}\ \left(\%\right) = 100\ \mathrm{x}\ \left(\mathrm{M}\mathrm{B}\ \hbox{--}\ \mathrm{M}\mathrm{C}\right)/\mathrm{M}\mathrm{B} $$

Where:

- MC is the geometric mean number of fleas on flea count day
- MB is the geometric mean number of live fleas count on baseline.

Note: Efficacy was calculated using both geometric and arithmetic means; however, geometric means were considered as the primary approach for effectiveness evaluation.

## Results

Thirty-six homes were originally enrolled in the study. However, two households (one in the fluralaner group and one in the afoxolaner group) did not remain in the study for at least 30 days; data from those households were not included. In the fluralaner group, the owner was not reliable in scheduling rechecks following initial treatment. The owner of the afoxolaner household brought puppies under 6 months of age into the home within three weeks of treatment. Several other homes and/or dogs did not complete the entire 12-week study but remained up to days 40–45. Data from those homes and dogs were included in data analysis up to the point that follow up was no longer possible. In the fluralaner group, one home (1 dog) was lost after the day 40–45 recheck because the owner was unreachable by phone and was not found at the residence. In the afoxolaner group one dog was lost prior to the day 54–60 after succumbing to apparent “heat stroke”. The other on study dog in the home completed the study. A home with two dogs treated with afoxolaner was lost on the last day of the study because the owner failed to appear for the appointment. A fourth afoxolaner dog was lost just prior to the last recheck when the owner moved out of the range of the study.

In the 17 homes in the fluralaner group that remained in the study beyond days 40–45 there were 34 dogs (avg. 15.1 kg; range 2.0–36.0 kg) enrolled. On day 0 dogs were treated in accordance with label direction and received a mean oral dose of 38.9 mg/kg (range 27.2–56.3 mg/kg) fluralaner. There were an additional 9 dogs in these homes that did not qualify for the study because they had an insufficient numbers of fleas (< 5) on day 0, resided permanently outdoors, were brought into the home after the enrollment date, or could not be safely handled by flea team members during flea counts. Therefore, there were a total of 43 dogs resident in the 17 homes that were treated with fluralaner on day 0.

In the 17 homes in the afoxolaner group that remained in the study beyond days 40–45, there were 27 dogs (avg. 16.7 kg; range 3.6–36.8 kg) enrolled in the study. On day 0 the dogs were treated in accordance with label direction and received a mean dose of 3.6 mg/kg (range 2.7–5.4 mg/kg) afoxolaner. Dogs in the afoxolaner group were similarly treated by study personnel two additional times; on day 28–30 and day 54–60. There were an additional 9 dogs in these homes that did not qualify for the study because of the same factors listed previously. Therefore, there were a total of 36 dogs resident in the 17 homes that were treated with afoxolaner on day 0.

On day 0, pets in the fluralaner treatment group had a geometric mean of 28.3 (range 5–155) fleas observed in area counts (Table 3). Pets in the afoxolaner treatment group had a geometric mean of 20.4 (range 5–178) fleas observed in area counts on day 0. The geometric mean flea count of fluralaner treated dogs was significantly higher on Day 0 than that of afoxolaner treated dogs (*P =* 0.004) Within 7 days of administration of fluralaner or afoxolaner the flea counts were reduced by 99.00 % and 99.3 %, respectively (Table 3). By days 28–30 the flea counts in the fluralaner treatment group were reduced by 100.0 % and reductions remained at 99.9 % to 100 % for the remainder of the 12-week study following a single oral dose on day 0 (Table 3). Afoxolaner also markedly reduced flea populations, with 99.9 % control on day 28–30 and 99.9 %–100 % control out to days 82–86 after 3 monthly doses. (Table 3). Both products significantly reduced flea counts on dogs at each time point (*P <*0.001). The flea counts of the two treatment groups were not significantly different on any of the post-treatment count days (*P* ≥ 0.327).

**Table 3:** On-animal flea counts in naturally infested homes when dogs were administered either fluralaner or afoxolaner oral treatments

Treatment group	# dogs at day 0		Days post-Treatment^a^							
			Day 0	Day 7	Day 14	Day 21	Day 28–30	Day 40–45	Day 54–60	Day 82–86
Fluralaner^b^	34	Geomean^d^	28.3	0.3	0.1	0.1	0.0	0.0	0.0	0.0
		range	(5–155)	(0–6)	(0–3)	(0–2)	(0–0)	(0–1)	(0–0)	(0–0)
		% control^e^		99.0	99.6	99.8	100.0	99.9	100.0	100.0
		% (#) dogs with no fleas	0.0 (0/34)	76.5 (26/34)	88.2 (30/34)	94.1 (32/34)	100.0 (34/34)	97.1 (33/34)	100.0 (33/33)	100.0 (33/33)
Afoxolaner^c^	27	Geomean	20.4	0.1	0.2	0.1	0.0	0.0	0.0	0.0
		range	(5–178)	(0–4)	(0–2)	(0–3)	(0–1)	(0–0)	(0–1)	(0–0)
		% control		99.3	99.2	99.6	99.9	100.0	99.9	100.0
		% (#) dogs with no fleas	0.0 (0/27)	85.2 (23/27)	81.5 (22/27)	92.6 (25/27)	96.3 (26/27)	100.0 (27/27)	96.2 (25/26)	100.0 (23/23)

^a^In both groups, dogs were treated on day 0. In the afoxolaner group, dogs were also treated once between days 28–30 and once between days 54–60

^b^Dogs were orally administered fluralaner chews (Bravecto® Merck Animal Health) according to label directions

^c^Dogs were orally administered afoxolaner chewables (NexGard® Merial, Inc.) according to label directions

^d^Geometric mean numbers of fleas in visual area counts on pets

^e^{(Day 0 geometric mean animal area flea counts—day x geometric mean animal area flea counts)/day 0 geometric mean animal area flea counts)} x 100

The number of flea free dogs in both groups following treatment were similar (Table 3). Following a single oral dose of fluralaner 88.2 % (30/34) of dogs were completely flea free by day 14 and only a single flea was found on one dog between days 28–30 and days 82–86, with all dogs flea free at the end of the study. Similarly 81.5 % (22/27) of the dogs in the afoxolaner treatment group were flea free by day 14 and only a single flea was found at two time points between days 28–30 and 82–86 following three monthly doses. All dogs were flea free by the end of the study (Table 3). Again, both products produced statistically significant more flea free dogs at each time point relative to study onset, (*P <*0.001) but there was no statistically significant difference in the number of flea free dogs between treatment groups (*P* ≥ 0.4407).

During the entire 12-week study, 1,267 fleas were trapped in the 34 residences using intermittent light traps and all were identified as *Ctenocephalides felis felis*, the cat flea. On day 0, the traps collected a geometric mean of 19.2 (range 6–122) and 14.0 (range 6–167) fleas in homes in the fluralaner and afoxolaner treatment groups, respectively (Table 4). Reductions in premises flea trap counts were 99.6 % and 100.0 % by days 28–30 and 82–86, respectively in the homes where pets were treated with fluralaner (Table 4). Reductions in premises flea trap counts were similar in homes where pets were treated with afoxolaner, with 96.7 % and 98.9 % by days 28–30 and 82–86, respectively (Table 4).

**Table 4:** Fleas recovered in premises flea traps in naturally infested homes when dogs were administered either fluralaner or afoxolaner chews

Treatment group	# homes		Days post-Treatment^a^							
			Day 0	Day 7	Day 14	Day 21	Day 28–30	Day 40–45	Day 54–60	Day 82–86
Fluralaner^b^	17	Geomean^d^	19.2	1.5	0.5	1.2	0.1	0.1	0.0	0.0
		% control^e^		92.4	97.3	93.9	99.6	99.7	99.8	100.0
		range	(6–122)	(0–8)	(0–5)	(0–4)	(0–1)	(0–2)	(0–1)	(0–0)
Afoxolaner^c^	17	Geomean	14.0	1.9	1.7	1.4	0.5	0.3	0.3	0.1
		% control		86.5	88.0	90.2	96.7	98.2	97.8	98.9
		range	(6–167)	(0–10)	(0–12)	(0–23)	(0–26)	(0–11)	(0–8)	(0–1)

^a^ In both groups, dogs were treated on day 0. In the afoxolaner group dogs were also treated once between days 28–30 and once between days 54–60

^b^Dogs were orally administered fluralaner chews (Bravecto® Merck Animal Health) according to label directions

^c^Dogs were orally administered afoxolaner chewables (NexGard® Merial, Inc.) according to label directions

^d^Geometric mean numbers of fleas recovered in two intermittent light flea traps averaged within households

^e^{(Day 0 geometric mean trap flea counts—day x geometric mean trap flea counts)/day 0 geometric mean trap flea counts)} x 100

Households without fleas on pets or in traps were considered flea free. Treatment with one dose of fluralaner resulted in 88.2 % (15/17) flea free households within 28–30 days of treatment and 100 % of households (16/16) free of fleas at week 12. Treatment with three monthly doses of afoxolaner resulted in 70.6 % (12/17) flea free households by day 28–30 and 80 % (12/15) of households free of fleas at week 12. There was no statistically significant difference in the number of flea free households between treatment groups (*P* ≥ 0.1012).

Dogs in the fluralaner and afoxolaner treatment groups had mean total flea bite hypersensitivity severity lesion scores of 12.8 (range 0–31) and 10.7 (range 1–30), respectively (Table 5) on day 0. By the end of the three-month study flea bite hypersensitivity severity lesion scores in the dogs were reduced to 1.8 (85.5 % improvement) and 2.0 (81.3 % improvement), in the fluralaner and afoxolaner treatment groups respectively (Table 5). Both products produced statistically significant reductions in signs of flea bite hypersensitivity relative to study onset, (*P <*0.001), but there was no statistically significant difference between treatment groups (*P* ≥ 0.087). Interestingly, CADESI-4 lesion scores were also similarly reduced. On day 0, dogs in the fluralaner and afoxolaner treatment groups had mean total CADESI-4 lesion scores of 51.5 (range 9–80) and 51.7 (range 3–91), respectively (Table 6). By the end of the three-month study CADESI-4 lesion scores in the dogs were reduced to 9.1 (82.3 % improvement) in the fluralaner treatment group and 8.7 (83.2 % improvement), in the afoxolaner treatment group (Table 6). Both products produced statistically significant reductions in CADESI-4 lesion scores relative to study onset, (*P <*0.001), but there was no statistically significant difference between treatment groups (*P ≥* 0.493).

**Table 5:** Assessment of skin lesions using a flea bite hypersensitivity severity scale for dogs naturally infested with fleas and administered either fluralaner or afoxolaner oral treatments

Treatment group		Days post-treatment^a^			
		Day 0	Day 28–30	Day 54–60	Day 82–86
Fluralaner^b^	# Dogs	34	34	33	33
	Mean Score^d^	12.8	5.4	3.7	1.8
	STDEV	8.22	4.13	3.80	1.64
	Range	(0–31)	(0–21)	(0–21)	(0–7)
	Reduction^e^		58.1 %	71.0 %	85.5 %
Afoxolaner^c^	# Dogs	27	27	26	23
	Mean Score^d^	10.7	4.9	2.7	2.0
	STDEV	7.15	3.35	2.76	2.30
	Range	(1–30)	(0–12)	(0–13)	(0–8)
	Reduction		54.0 %	74.5 %	81.3 %

^a^In both groups dogs were treated on day 0. In the afoxolaner group dogs were also treated once between days 28–30 and once between days 54–60

^b^Dogs were orally administered fluralaner chews (Bravecto® Merck Animal Health) according to label directions

^c^Dogs were orally administered afoxolaner chewables (NexGard® Merial, Inc.) according to label directions

^d^Arithmetic mean Flea Allergy Dermatitis lesion score (Wilkerson et al. Vet Immunol Immunopathol 99(3–4):179–192, 2004)

^e^{(Day 0 arithmetic mean score—day x arithmetic mean score)/day 0 arithmetic mean score)} x 100

**Table 6:** Assessment of skin lesions using the Canine Atopic Dermatitis Extent and Severity Index-4 (CADESI-4) scale for dogs naturally infested with fleas and administered either fluralaner or afoxolaner oral treatments

Treatment group		Days post-Treatment^a^			
		Day 0	Day 28–30	Day 54–60	Day 82–86
Fluralaner^b^	# Dogs	34	34	33	33
	Mean CADESI - 4 Score^d^	51.5	20.4	15.2	9.1
	STDEV	17.53	8.99	9.95	5.29
	Range	(9–80)	(0–39)	(0–40)	(0–20)
	Reduction^e^		60.4 %	70.5 %	82.3 %
Afoxolaner^c^	# Dogs	27	27	26	23
	Mean CADESI - 4 Score^d^	51.7	18.8	12.7	8.7
	STDEV	22.28	14.80	12.51	7.70
	Range	(3–91)	(3–51)	(0–48)	(0–26)
	Reduction^e^		63.6 %	75.4 %	83.2 %

^a^In both groups dogs were treated on day 0. In the afoxolaner group, dogs were also treated once between days 28–30 and once between days 54–60

^b^Dogs were orally administered fluralaner chews (Bravecto® Merck Animal Health) according to label directions

^c^Dogs were orally administered afoxolaner chewables (NexGard® Merial, Inc.) according to label directions

^d^Arithmetic mean Canine Atopic Dermatitis Extent and Severity Index (CADESI)-4 scores (Olivry et al. Vet Dermatol 25(2):77–85, 2014)

^e^{(Day 0 arithmetic mean CADESI-4 score – day x arithmetic mean CADESI-4 score)/day 0 arithmetic mean CADESI-4 score)} x 100

Owner assessed pruritus scores showed rapid and marked improvement in both treatment groups. On day 0, dogs in the fluralaner and afoxolaner treatment groups had mean pruritus visual analogue scale (PVAS) scores of 7.3 (range 2.2–10) and 7.1 (range 2.7–10), respectively (Table 6). Within 2 weeks post-treatment, mean pruritus scores had dropped by 71.0 % (2.1) and 73.8 % (1.9) in the fluralaner and afoxolaner treatment groups, respectively (Table 7). By the end of the study the mean pruritus scores in both groups had fallen to 0.9 and 0.7, in the fluralaner and afoxolaner treatment groups, respectively (Table 7). Both products produced statistically significant reductions in PVAS scores relative to study onset, (*P <*0.001), but there was no statistically significant difference between treatment groups (*P* ≥ 0.695).

**Table 7:** Owner assessment of pruritus using a visual analogue scale (PVAS) for dogs naturally infested with fleas and administered either fluralaner or afoxolaner treatments

Treatment group		Days post-Treatment^a^			
		Day 0	Day 28–30	Day 54–60	Day 82–86
Fluralaner ^b^	# Dogs	34	34	33	33
	Mean PVAS Score^d^	7.3	1.7	0.9	0.9
	STDEV	2.40	1.8	1.2	1.3
	Range	(2.2–10)	(0-6)	(0-4.2)	(0-4.2)
	Reduction^e^		77.0 %	87.5 %	88.0 %
Afoxolaner ^c^	# Dogs	27	27	26	23
	Mean PVAS Score^d^	7.1	1.5	0.7	0.7
	STDEV	2.2	1.8	0.9	1.4
	Range	(2.7–10)	(0-6.5)	(0-3.6)	(0-6.7)
	Reduction		79.2 %	89.6 %	89.7 %

^a^In both groups dogs were treated on day 0. In the afoxolaner group dogs were also treated once between days 28–30 and once between days 54–60

^b^Dogs were orally administered fluralaner chews (Bravecto® Merck Animal Health) according to label directions

^c^Dogs were orally administered afoxolaner chewables (NexGard® Merial, Inc.) according to label directions

^d^Arithmetic mean pruritus score as assessed by dog owners using the PVAS (Hill et al. Vet Dermatol 18(5):301–308, 2004)

^e^{(Day 0 arithmetic mean FAD score—day x arithmetic mean FAD score)/day 0 arithmetic mean FAD score)} x 100

Nine adverse events were reported in treated dogs in both groups during the course of the trial. In the fluralaner treatment group, one owner reported that a dog was lethargic for 1 to 2 days following fluralaner administration, and was acting normally on the 3^rd^ day. Another owner reported that a fluralaner treated dog was lethargic and had increased pruritus for a few days during the week following treatment, and was acting normally before the 7 day recheck. Another owner reported that 27 days after fluralaner treatment, his 2 dogs ate grass and vomited. Another owner reported that 2 of the 5 fluralaner treated dogs in the home had periodic episodes of coughing during the study. This owner declined offer of diagnostic evaluation of the two dogs.

In the afoxolaner treatment group an owner reported that their dog vomited 27 days after treatment, and appeared normal and healthy at retreatment on day 29. Another owner reported loose stools in an afoxolaner treated dog from day 9 through day 13. One owner reported that one of five afoxolaner treated dogs in the household died prior to the day 54–60 evaluation. The owner assessed the cause as “heat stroke” when the air-conditioner broke during the day while the owner was absent. The owner disposed of the dog’s body prior to informing the study investigators, and it was not possible to conduct a necropsy.

## Discussion

Fluralaner chews provided excellent flea control achieving 99.0 % reduction in geometric mean area flea counts on dogs within one week and complete elimination of on-animal flea burdens within 2 months after a single dosage. There were still no fleas on any dog and none in any home at the end of the 12-week study. The residual activity of this formulation was remarkable given the potential for continual flea infestation pressure from infested indoor and outdoor premises. The afoxolaner chewables administered monthly also provided good flea control. Following three monthly oral doses of afoxolaner the geometric mean area flea counts on dogs was also reduced by 100 % by the end of the 12 week study and > 80 % of homes were flea free. The level of efficacy observed in this study for the afoxolaner chewable was very similar to that observed in a previous field study conducted in west central Florida (Tampa Bay) [11].

The area count technique used in this and previous in-home investigations in west central Florida has been shown to detect an average of 23.5 % of the total pet flea burden [29]. Therefore, average pretreatment total body flea burdens of dogs in the fluralaner and afoxolaner treatment groups based on geometric means area counts of 28.3 and 20.4 can be estimated to be approximately 120 and 86.8 for dogs in this study, respectively.

It is important to note that in previous in-home studies conducted in west central Florida the percent reductions in on-animal flea counts were often less than 100 % even through 90 days of treatment [3–8, 10]. In a 2010 study where dogs were treated with either a dinotefuran-pyriproxyfen or fipronil (s)-methoprene topical spot-on formulation, only 60.0 % and 55.6 % of dogs were completely flea free by days 54–60 [8]. In 2013 even though the efficacy against on-animal flea burdens of monthly topical indoxacarb was 99.1 %, the number of completely flea free dogs was only 77.1 %, by the end of the two month study [10]. In that 2013 study, the monthly application of fipronil (s)-methoprene topical spot-on only achieved 15.6 % flea free status by 2 months.

In contrast to these previous studies, when dogs were administered either fluralaner or afoxolaner oral treatments, 88.2 % and 81.5 % were flea free within 2 weeks and 100 % and 96.3 % were flea free within 4 weeks, respectively. These data combined with the on-animal flea count efficacy data are indicating that these isoxazolines have a very rapid residual speed of kill under natural flea exposure conditions. Previous reports from a laboratory study suggest minor differences in the speed of kill may have clinical consequences [26]. However, this real world field study demonstrated no statistically significant differences between one dose of fluralaner and three doses of afoxolaner in any of the flea infestation or clinical parameters assessed. In addition, the continued residual efficacy of a single oral dose of fluralaner was evidenced by the fact that from evaluations conducted between days 28–30 post-treatment up to and including evaluations on week 12, only a single flea was ever found on a single dog. Furthermore, one administration of fluralaner per dog cleared all households completely of fleas in the 12 weeks of this study.

In this current study one of the objectives was to assess the effect of these treatments on reducing dermatitis and pruritus in treated dogs. Flea allergy dermatitis and atopic dermatitis are the most common allergic skin diseases in dogs and cats [34]. To evaluate flea allergy dermatitis we chose a flea bite hypersensitivity lesional scoring system that was previously published from a laboratory controlled FAD induction study [32]. Although multiple validated scoring systems for canine atopic dermatitis exist, currently, the International Committee on Allergic Diseases of Animals (ICADA) recommends CADESI-4, or, canine atopic dermatitis lesion index (CADLI) as the only two validated severity scales to score skin lesions of canine atopic dermatitis patients [33]. CADESI-4 was utilized in this study for a more thorough dermatologic lesion assessment. It is important to note that a definitive diagnosis of FAD or CAD was not attained for the patients enrolled in the study. The intent was to have a single investigator clinically evaluate each of the study participants utilizing the aforementioned scoring systems to determine the clinical relevance of flea control measures when additional treatments were not provided.

The owner reported pruritus assessment tool (PVAS) we used was previously validated and used in field trials [9, 30, 31]. Pruritus is a subjective parameter and is important in evaluating response to therapy in a patient with pruritic dermatitis [30, 31]. There was rapid statistically significant improvement in PVAS scores for patients receiving fluralaner or afoxolaner despite no concurrent therapies.

The diagnosis of flea allergy dermatitis is made based on history, clinical signs, and response to anti-flea therapy [35]. Intradermal allergy testing and serological testing can have variable results and are not recommended for diagnosis of flea allergy on their own. Specificity, sensitivity, and accuracy of intradermal testing with whole-body flea extracts can vary, especially if performed in a flea endemic region [35]. Definitive diagnosis of flea allergy dermatitis must be made by the resolution of clinical signs in response to flea control and the recurrence of those signs upon re-exposure to fleas. In this current study there was statistically significant improvement in the flea bite hypersensitivity severity scores in both treatment groups. By days 54–60, there was 85.5 % and 81.3 % improvement with fluralaner and afoxolaner, respectively. The marked improvement in PVAS as well as the FAD scores upon treatments with fluralaner and afoxolaner supports that most of the participants had clinical signs attributable to flea exposure.

Diagnosis of canine atopic dermatitis is mainly a diagnosis of exclusion that requires a systematic approach [36, 37]. Diagnosis is based on history, clinical signs, and ruling out other pruritic diseases that share similar characteristics [36, 37]. Many of the study participants had lesions consistent with those that can be seen in atopic dermatitis, however further evaluation would be necessary for this diagnosis to be made. Dogs exhibited clinical signs consistent with atopic dermatitis, though it was beyond this study to pursue a more definitive diagnosis. The improvement of CADESI-4 scores, could support the concept that there is a threshold for the development of atopic dermatitis. The concept of threshold for the development of atopic dermatitis relates to the allergen load. When the allergen load is low, no symptoms may be observed, however when a heavy allergen burden exists, clinical disease results [34]. In our study, the elimination of fleas without the benefit of additional treatments caused statistically significant improvement in PVAS, CADESI-4, and FAD scores. Therefore, it is possible that the elimination of fleas resulted in a reduction of stimuli below the pruritic threshold, and/or, that the allergen burden was reduced such that if participants were atopic dogs, they were able to experience significant improvement [34].

An adverse event is any clinical sign reported following treatment administration regardless of causality. Adverse events were reported following administration of both fluralaner and afoxolaner in the course of this study. The reported events in this study may have been treatment associated or may have been independent of treatment administration. All but one of the reports were considered to be not serious and all of these resolved without treatment, with none of the affected dogs withdrawn from the study. The afoxolaner treated dog that died was unfortunately not available for necropsy examination.

Despite study protocol requirements that dogs not be pregnant, breeding or lactating during the 84-day study, one dog in the fluralaner treated group did become pregnant and delivered puppies on study day 49. As fluralaner is approved for pregnant and lactating dogs, this dog continued in the study and birthed a healthy litter of puppies.

## Conclusions

This clinical field investigation conducted during the summer of 2015 in subtropical west central Florida clearly demonstrated that these oral isoxazoline formulations rapidly and effectively controlled flea populations in homes and on dogs. While the afoxolaner chewable was administered three times at monthly intervals, fluralaner eliminated fleas on dogs and in the homes within 12 weeks following a single dose. Not only were flea populations controlled by these formulations, but marked improvement in dermatologic lesions and pruritus were also observed without the use of any concurrent medical intervention.

## Abbreviations

AIC, Akaike’s information criterion; CAD, canine atopic dermatitis; CADESI, canine atopic dermatitis extent and severity index scoring system; CADLI, canine atopic dermatitis lesion index; FAD, flea allergy dermatitis; ICADA, international committee on allergic diseases of Animals; PVAS, pruritus visual analogue scale
